# Correction: Pérez et al. Biomarker-Based Nomogram to Predict Neoadjuvant Chemotherapy Response in Muscle-Invasive Bladder Cancer. *Biomedicines* 2025, *13*, 740

**DOI:** 10.3390/biomedicines14071604

**Published:** 2026-07-17

**Authors:** Meritxell Pérez, Juan José Lozano, Mercedes Ingelmo-Torres, Montserrat Domenech, Caterina Fernández Ramón, J. Alfred Witjes, Antoine G. van der Heijden, Maria José Requena, Antonio Coy, Ricard Calderon, Begoña Mellado, Antonio Alcaraz, Antoni Vilaseca, Maria J. Ribal

**Affiliations:** 1 Department of Urology, Hospital Universitari Terrassa, 08221 Terrassa, Spain; mperez@cst.cat; 2 Departament de Cirurgia i Especialitats Medicoquirúrgiques, Facultat de Medicina i Ciències de la Salut, Universitat de Barcelona (UB), c. Casanova, 143, 08036 Barcelona, Spain; 3 Plataforma Bioinformatica, Centro de Investigación Biomédica en Red Enfermedades Hepáticas y Digestivas (CIBERehd), Hospital Clínic de Barcelona, 08036 Barcelona, Spain; 4 Department of Urology, Institut d’Investigacions Biomèdiques August Pi i Sunyer (IDIBAPS), Hospital Clínic de Barcelona, University of Barcelona, 08036 Barcelona, Spain; 5 Medical Oncology Department, Fundació Althaia, Xarxa Assintencial Universitària de Manresa, 08242 Manresa, Spain; 6 Urology Department, Fundació Althaia, Xarxa Assintencial Universitària de Manresa, 08242 Manresa, Spain; 7 Department of Urology, Radboud University Medical Center, 6525 GA Nijmegen, The Netherlands; 8 Department of Urology, Reina Sofía University Hospital, IMIBIC, Cordoba University, 14014 Córdoba, Spain; 9 Fundación Instituto Valenciano de Oncologia (IVO), 46009 Valencia, Spain; 10 Translational Genomics and Targeted Therapeutics in Solid Tumors, Medical Oncology Department, August Pi i Sunyer Biomedical Research Institute (IDIBAPS), Hospital Clínic de Barcelona, 08036 Barcelona, Spain; 11 Uro-Oncology Unit, Medical Oncology Department, Hospital Clínic de Barcelona, University of Barcelona, 08036 Barcelona, Spain; 12 Uro-Oncology Unit, Department of Urology, Hospital Clinic de Barcelona, 08036 Barcelona, Spain

## Additional Affiliation

In the published publication [1], there was an error regarding the affiliation for Meritxell Pérez. In addition to affiliation 1, the updated affiliation should include: Departament de Cirurgia i Especialitats Medicoquirúrgiques, Facultat de Medicina i Ciències de la Salut, Universitat de Barcelona (UB), c. Casanova, 143, 08036 Barcelona, Spain. The authors state that the scientific conclusions are unaffected. This correction was approved by the Academic Editor. The original publication has also been updated.
